# Supplementary material for: Variations and gradients between methane seep and off-seep microbial communities in a submarine canyon system in the Northeast Pacific
Source: PeerJ. 2023 Mar 28;11:e15119. doi: 10.7717/peerj.15119 (PMC10064993; doi:10.7717/peerj.15119)
Supplement: Supplemental Information 2 — Calculated from rarefied data, see Methods. [file peerj-11-15119-s002.docx]

***Supplemental Table 1 – Shannon alpha-diversity metrics for samples.*** Calculated from rarefied data, see Methods.

| **Sample** | **Site** | **depth in sediment (cm)** | **Shannon** |
| --- | --- | --- | --- |
| CanyonA | Quinault_Canyon | 1 | 7.24508 |
| CanyonA | Quinault_Canyon | 2 | 7.695325 |
| CanyonA | Quinault_Canyon | 3 | 7.259301 |
| CanyonA | Quinault_Canyon | 4 | 7.165717 |
| CanyonA | Quinault_Canyon | 5 | 7.14894 |
| CanyonA | Quinault_Canyon | 6 | 7.031014 |
| CanyonA | Quinault_Canyon | 7 | 7.163505 |
| CanyonA | Quinault_Canyon | 8 | 7.094623 |
| CanyonA | Quinault_Canyon | 9 | 6.803228 |
| CanyonA | Quinault_Canyon | 10 | 7.30012 |
| Dagorlad_150m | Dagorlad_Seep | 1 | 6.114587 |
| Dagorlad_150m | Dagorlad_Seep | 2 | 6.276029 |
| Dagorlad_150m | Dagorlad_Seep | 3 | 6.307875 |
| Dagorlad_150m | Dagorlad_Seep | 4 | 6.409749 |
| Dagorlad_150m | Dagorlad_Seep | 5 | 6.456318 |
| Dagorlad_150m | Dagorlad_Seep | 6 | 6.351906 |
| Dagorlad_150m | Dagorlad_Seep | 7 | 6.335207 |
| Dagorlad_150m | Dagorlad_Seep | 8 | 6.217446 |
| Dagorlad_150m | Dagorlad_Seep | 9 | 6.170209 |
| Dagorlad_150m | Dagorlad_Seep | 10 | 6.037075 |
| Dagorlad_50m | Dagorlad_Seep | 1 | 6.512842 |
| Dagorlad_50m | Dagorlad_Seep | 2 | 6.433979 |
| Dagorlad_50m | Dagorlad_Seep | 3 | 5.364734 |
| Dagorlad_50m | Dagorlad_Seep | 4 | 6.084487 |
| Dagorlad_50m | Dagorlad_Seep | 5 | 6.606573 |
| Dagorlad_50m | Dagorlad_Seep | 6 | 6.370519 |
| Dagorlad_50m | Dagorlad_Seep | 7 | 6.28054 |
| Dagorlad_50m | Dagorlad_Seep | 8 | 6.162246 |
| Dagorlad_50m | Dagorlad_Seep | 9 | 6.246016 |
| Dagorlad_50m | Dagorlad_Seep | 10 | 6.366828 |
| Dagorlad_5m | Dagorlad_Seep | 1 | 6.326002 |
| Dagorlad_5m | Dagorlad_Seep | 2 | 6.695544 |
| Dagorlad_5m | Dagorlad_Seep | 3 | 6.235752 |
| Dagorlad_5m | Dagorlad_Seep | 4 | 6.366143 |
| Dagorlad_5m | Dagorlad_Seep | 5 | 6.374536 |
| Dagorlad_5m | Dagorlad_Seep | 6 | 6.630269 |
| Dagorlad_5m | Dagorlad_Seep | 7 | 6.402182 |
| Dagorlad_5m | Dagorlad_Seep | 8 | 6.460002 |
| Dagorlad_5m | Dagorlad_Seep | 9 | 6.775118 |
| Dagorlad_5m | Dagorlad_Seep | 10 | 5.929584 |
| Dagorlad_Clams | Dagorlad_Seep | 1 | 5.652453 |
| Dagorlad_Clams | Dagorlad_Seep | 2 | 6.36265 |
| Dagorlad_Clams | Dagorlad_Seep | 3 | 5.254307 |
| Dagorlad_Clams | Dagorlad_Seep | 4 | 5.457829 |
| Dagorlad_Clams | Dagorlad_Seep | 5 | 6.245262 |
| Dagorlad_Clams | Dagorlad_Seep | 6 | 5.7736 |
| Dagorlad_Clams | Dagorlad_Seep | 7 | 5.394069 |
| Dagorlad_Clams | Dagorlad_Seep | 8 | 5.651868 |
| Dagorlad_Clams | Dagorlad_Seep | 9 | 5.296132 |
| Dagorlad_Clams | Dagorlad_Seep | 10 | 5.399798 |
| Dagorlad_MatA | Dagorlad_Seep | 1 | 4.945343 |
| Dagorlad_MatA | Dagorlad_Seep | 2 | 5.284979 |
| Dagorlad_MatA | Dagorlad_Seep | 3 | 5.01186 |
| Dagorlad_MatA | Dagorlad_Seep | 4 | 5.35962 |
| Dagorlad_MatA | Dagorlad_Seep | 5 | 5.494235 |
| Dagorlad_MatA | Dagorlad_Seep | 6 | 5.786904 |
| Dagorlad_MatA | Dagorlad_Seep | 7 | 5.331852 |
| Dagorlad_MatA | Dagorlad_Seep | 8 | 5.332466 |
| Dagorlad_MatA | Dagorlad_Seep | 9 | 5.317271 |
| Dagorlad_MatA | Dagorlad_Seep | 10 | 4.901652 |
| Emyn_Muil_150m | Emyn_Muil_Seep | 1 | 6.941736 |
| Emyn_Muil_150m | Emyn_Muil_Seep | 2 | 7.403911 |
| Emyn_Muil_150m | Emyn_Muil_Seep | 3 | 5.271524 |
| Emyn_Muil_150m | Emyn_Muil_Seep | 4 | 7.300292 |
| Emyn_Muil_150m | Emyn_Muil_Seep | 5 | 7.384743 |
| Emyn_Muil_150m | Emyn_Muil_Seep | 6 | 7.205588 |
| Emyn_Muil_150m | Emyn_Muil_Seep | 7 | 7.24265 |
| Emyn_Muil_150m | Emyn_Muil_Seep | 8 | 7.17154 |
| Emyn_Muil_150m | Emyn_Muil_Seep | 9 | 6.206748 |
| Emyn_Muil_150m | Emyn_Muil_Seep | 10 | 4.901347 |
| Emyn_Muil_50m | Emyn_Muil_Seep | 1 | 5.160592 |
| Emyn_Muil_50m | Emyn_Muil_Seep | 2 | 5.280754 |
| Emyn_Muil_50m | Emyn_Muil_Seep | 3 | 5.250987 |
| Emyn_Muil_50m | Emyn_Muil_Seep | 4 | 5.267828 |
| Emyn_Muil_50m | Emyn_Muil_Seep | 5 | 5.381469 |
| Emyn_Muil_50m | Emyn_Muil_Seep | 6 | 5.267022 |
| Emyn_Muil_50m | Emyn_Muil_Seep | 7 | 5.184539 |
| Emyn_Muil_50m | Emyn_Muil_Seep | 8 | 5.242 |
| Emyn_Muil_50m | Emyn_Muil_Seep | 9 | 5.260749 |
| Emyn_Muil_50m | Emyn_Muil_Seep | 10 | 5.228058 |
| Emyn_Muil_5m | Emyn_Muil_Seep | 1 | 6.34566 |
| Emyn_Muil_5m | Emyn_Muil_Seep | 2 | 6.64053 |
| Emyn_Muil_5m | Emyn_Muil_Seep | 3 | 6.505957 |
| Emyn_Muil_5m | Emyn_Muil_Seep | 4 | 6.286415 |
| Emyn_Muil_5m | Emyn_Muil_Seep | 5 | 6.470553 |
| Emyn_Muil_5m | Emyn_Muil_Seep | 6 | 6.592633 |
| Emyn_Muil_5m | Emyn_Muil_Seep | 7 | 6.34036 |
| Emyn_Muil_5m | Emyn_Muil_Seep | 8 | 5.817783 |
| Emyn_Muil_5m | Emyn_Muil_Seep | 9 | 5.329403 |
| Emyn_Muil_5m | Emyn_Muil_Seep | 10 | 6.460065 |
| Emyn_Muil_MatA | Emyn_Muil_Seep | 1 | 6.743913 |
| Emyn_Muil_MatA | Emyn_Muil_Seep | 2 | 6.023313 |
| Emyn_Muil_MatA | Emyn_Muil_Seep | 3 | 5.908465 |
| Emyn_Muil_MatA | Emyn_Muil_Seep | 4 | 6.038111 |
| Emyn_Muil_MatA | Emyn_Muil_Seep | 5 | 5.988297 |
| Emyn_Muil_MatA | Emyn_Muil_Seep | 6 | 5.956675 |
| Emyn_Muil_MatA | Emyn_Muil_Seep | 7 | 5.535345 |
| Emyn_Muil_MatA | Emyn_Muil_Seep | 8 | 4.879496 |
| Emyn_Muil_MatA | Emyn_Muil_Seep | 9 | 5.794601 |
| Emyn_Muil_MatA | Emyn_Muil_Seep | 10 | 6.113738 |
| Emyn_Muil_Worms | Emyn_Muil_Seep | 1 | 5.201673 |
| Emyn_Muil_Worms | Emyn_Muil_Seep | 2 | 4.804331 |
| Emyn_Muil_Worms | Emyn_Muil_Seep | 3 | 5.320909 |
| Emyn_Muil_Worms | Emyn_Muil_Seep | 4 | 5.212415 |
| Emyn_Muil_Worms | Emyn_Muil_Seep | 5 | 4.931941 |
| Emyn_Muil_Worms | Emyn_Muil_Seep | 6 | 5.095498 |
| Emyn_Muil_Worms | Emyn_Muil_Seep | 7 | 5.463522 |
| Emyn_Muil_Worms | Emyn_Muil_Seep | 8 | 5.121799 |
| Emyn_Muil_Worms | Emyn_Muil_Seep | 9 | 5.34077 |
| Emyn_Muil_Worms | Emyn_Muil_Seep | 10 | 5.005609 |
| OOI | OOI_Slope_Base | 1 | 5.920528 |
| OOI | OOI_Slope_Base | 2 | 5.703628 |
| OOI | OOI_Slope_Base | 3 | 5.780502 |
| OOI | OOI_Slope_Base | 4 | 5.742494 |
| OOI | OOI_Slope_Base | 5 | 5.741235 |
| OOI | OOI_Slope_Base | 6 | 5.71883 |
| OOI | OOI_Slope_Base | 7 | 5.68322 |
| OOI | OOI_Slope_Base | 8 | 5.982467 |
| OOI | OOI_Slope_Base | 9 | 6.106936 |
| OOI | OOI_Slope_Base | 10 | 5.830439 |
| Quinault_Shallow_180m | Quinault_500m | 1 | 7.126608 |
| Quinault_Shallow_180m | Quinault_500m | 2 | 6.98322 |
| Quinault_Shallow_180m | Quinault_500m | 3 | 7.103663 |
| Quinault_Shallow_180m | Quinault_500m | 4 | 7.177614 |
| Quinault_Shallow_180m | Quinault_500m | 5 | 7.108438 |
| Quinault_Shallow_180m | Quinault_500m | 6 | 6.389132 |
| Quinault_Shallow_180m | Quinault_500m | 7 | 5.825944 |
| Quinault_Shallow_180m | Quinault_500m | 8 | 6.495504 |
| Quinault_Shallow_180m | Quinault_500m | 9 | 6.781101 |
| Quinault_Shallow_180m | Quinault_500m | 10 | 6.599032 |
| Quinault_Shallow_50m | Quinault_500m | 1 | 7.225997 |
| Quinault_Shallow_50m | Quinault_500m | 2 | 5.755806 |
| Quinault_Shallow_50m | Quinault_500m | 3 | 6.19221 |
| Quinault_Shallow_50m | Quinault_500m | 4 | 6.205918 |
| Quinault_Shallow_50m | Quinault_500m | 5 | 6.024242 |
| Quinault_Shallow_50m | Quinault_500m | 6 | 5.880849 |
| Quinault_Shallow_50m | Quinault_500m | 7 | 5.602886 |
| Quinault_Shallow_50m | Quinault_500m | 8 | 5.638257 |
| Quinault_Shallow_50m | Quinault_500m | 9 | 5.608077 |
| Quinault_Shallow_50m | Quinault_500m | 10 | 5.540707 |
| Westmarch_ClamsA | Westmarch_Seep | 1 | 6.508361 |
| Westmarch_ClamsA | Westmarch_Seep | 2 | 6.642881 |
| Westmarch_ClamsA | Westmarch_Seep | 3 | 6.632728 |
| Westmarch_ClamsA | Westmarch_Seep | 4 | 6.654434 |
| Westmarch_ClamsA | Westmarch_Seep | 5 | 6.684123 |
| Westmarch_ClamsA | Westmarch_Seep | 6 | 6.745661 |
| Westmarch_ClamsA | Westmarch_Seep | 7 | 6.791625 |
| Westmarch_ClamsA | Westmarch_Seep | 8 | 6.651257 |
| Westmarch_ClamsA | Westmarch_Seep | 9 | 6.94247 |
| Westmarch_ClamsA | Westmarch_Seep | 10 | 6.783437 |
