# Supplementary material for: Variations and gradients between methane seep and off-seep microbial communities in a submarine canyon system in the Northeast Pacific
Source: PeerJ. 2023 Mar 28;11:e15119. doi: 10.7717/peerj.15119 (PMC10064993; doi:10.7717/peerj.15119)
Supplement: Supplemental Information 3 [file peerj-11-15119-s003.docx]

### Supplemental Table 2 - Database of genes for PICRUSt2 analysis.

| **KO** | **Category** | **Process** | **Gene** | **EC number** |
| --- | --- | --- | --- | --- |
| K00125 | Methane | methanogenesis | fdhB;formatedehydrogenase(coenzymeF420)betasubunit | EC:1.17.98.31.8.98.6 |
| K00200 | Methane | methanogenesis | fwdA,fmdA;formylmethanofurandehydrogenasesubunitA | EC:1.2.7.12 |
| K00201 | Methane | methanogenesis | fwdB,fmdB;formylmethanofurandehydrogenasesubunitB | EC:1.2.7.12 |
| K00202 | Methane | methanogenesis | fwdC,fmdC;formylmethanofurandehydrogenasesubunitC | EC:1.2.7.12 |
| K00203 | Methane | methanogenesis | fwdD,fmdD;formylmethanofurandehydrogenasesubunitD | EC:1.2.7.12 |
| K00204 | Methane | methanogenesis | fwdH;4Fe-4Sferredoxin |  |
| K00205 | Methane | methanogenesis | fwdF,fmdF;4Fe-4Sferredoxin |  |
| K00216 | Metals | siderophore | entA;2,3-dihydro-2,3-dihydroxybenzoatedehydrogenase | EC:1.3.1.28 |
| K00319 | Methane | methanogenesis | mtd;methylenetetrahydromethanopterindehydrogenase | EC:1.5.98.1 |
| K00320 | Methane | methanogenesis | mer;5,10-methylenetetrahydromethanopterinreductase | EC:1.5.98.2 |
| K00360 | Nitrogen | assimilatory nitrate reduction | nasB;assimilatorynitratereductaseelectrontransfersubunit | EC:1.7.99.- |
| K00362 | Nitrogen | dissimilatory nitrate reduction | nirB;nitritereductase(NADH)largesubunit | EC:1.7.1.15 |
| K00363 | Nitrogen | dissimilatory nitrate reduction | nirD;nitritereductase(NADH)smallsubunit | EC:1.7.1.15 |
| K00366 | Nitrogen | assimilatory nitrate reduction | nirA;ferredoxin-nitritereductase | EC:1.7.7.1 |
| K00367 | Nitrogen | assimilatory nitrate reduction | narB;ferredoxin-nitratereductase | EC:1.7.7.2 |
| K00368 | Nitrogen | denitrification | nirK;nitritereductase(NO-forming) | EC:1.7.2.1 |
| K00370 | Nitrogen | nitrate reduction | narG,narZ,nxrA;nitratereductase/nitriteoxidoreductase,alpha |  |
| K00371 | Nitrogen | nitrate reduction | narH,narY,nxrB;nitratereductase/nitriteoxidoreductase,beta |  |
| K00372 | Nitrogen | assimilatory nitrate reduction | nasA;assimilatorynitratereductasecatalyticsubunit | EC:1.7.99.- |
| K00374 | Nitrogen | nitrate reduction | narI,narV;nitratereductasegammasubunit | EC:1.7.5.11.7.99.- |
| K00376 | Nitrogen | nitrate reduction | nosZ;nitrous-oxidereductase | EC:1.7.2.4 |
| K00380 | Sulfur | assimilatory sulfate reduction | cysJ;sulfitereductase(NADPH)flavoproteinalpha-component | EC:1.8.1.2 |
| K00381 | Sulfur | assimilatory sulfate reduction | cysI;sulfitereductase(NADPH)hemoproteinbeta-component | EC:1.8.1.2 |
| K00385 | Sulfur | sulfite reduction | asrC;anaerobicsulfitereductasesubunitC |  |
| K00387 | Sulfur | sulfite oxidation | SUOX;sulfiteoxidase | EC:1.8.3.1 |
| K00390 | Sulfur | assimilatory sulfate reduction | cysH;phosphoadenosinephosphosulfatereductase | EC:1.8.4.81.8.4.10 |
| K00392 | Sulfur | assimilatory sulfate reduction | sir;sulfitereductase(ferredoxin) | EC:1.8.7.1 |
| K00394 | Sulfur | dissimilatory sulfate reduction | aprA;adenylylsulfatereductase,subunitA | EC:1.8.99.2 |
| K00395 | Sulfur | dissimilatory sulfate reduction | aprB;adenylylsulfatereductase,subunitB | EC:1.8.99.2 |
| K00399 | Methane | methanogenesis/methanotrophy | mcrA;methyl-coenzymeMreductasealphasubunit | EC:2.8.4.1 |
| K00401 | Methane | methanogenesis/methanotrophy | mcrB;methyl-coenzymeMreductasebetasubunit | EC:2.8.4.1 |
| K00402 | Methane | methanogenesis/methanotrophy | mcrG;methyl-coenzymeMreductasegammasubunit | EC:2.8.4.1 |
| K00531 | Nitrogen | nitrogen fixation | anfG;nitrogenasedeltasubunit | EC:1.18.6.1 |
| K00577 | Methane | methanogenesis | mtrA;tetrahydromethanopterinS-methyltransferasesubunitA | EC:2.1.1.86 |
| K00578 | Methane | methanogenesis | mtrB;tetrahydromethanopterinS-methyltransferasesubunitB | EC:2.1.1.86 |
| K00579 | Methane | methanogenesis | mtrC;tetrahydromethanopterinS-methyltransferasesubunitC | EC:2.1.1.86 |
| K00580 | Methane | methanogenesis | mtrD;tetrahydromethanopterinS-methyltransferasesubunitD | EC:2.1.1.86 |
| K00581 | Methane | methanogenesis | mtrE;tetrahydromethanopterinS-methyltransferasesubunitE | EC:2.1.1.86 |
| K00582 | Methane | methanogenesis | mtrF;tetrahydromethanopterinS-methyltransferasesubunitF | EC:2.1.1.86 |
| K00583 | Methane | methanogenesis | mtrG;tetrahydromethanopterinS-methyltransferasesubunitG | EC:2.1.1.86 |
| K00584 | Methane | methanogenesis | mtrH;tetrahydromethanopterinS-methyltransferasesubunitH | EC:2.1.1.86 |
| K00672 | Methane | methanogenesis | ftr;formylmethanofuran--tetrahydromethanopterinN-formyltransferase | EC:2.3.1.101 |
| K00860 | Sulfur | assimilatory sulfate reduction | cysC;adenylylsulfatekinase | EC:2.7.1.25 |
| K00955 | Sulfur | assimilatory sulfate reduction | cysNC;bifunctionalenzymeCysN/CysC | EC:2.7.7.42.7.1.25 |
| K00956 | Sulfur | assimilatory sulfate reduction | cysN;sulfateadenylyltransferasesubunit1 | EC:2.7.7.4 |
| K00957 | Sulfur | assimilatory sulfate reduction | cysD;sulfateadenylyltransferasesubunit2 | EC:2.7.7.4 |
| K00958 | Sulfur | sulfate reduction | sat,met3;sulfateadenylyltransferase | EC:2.7.7.4 |
| K01252 | Metals | siderophore | entB,dhbB,vibB,mxcF;bifunctionalisochorismatelyase/aryl |  |
| K01499 | Methane | methanogenesis | mch;methenyltetrahydromethanopterincyclohydrolase | EC:3.5.4.27 |
| K01725 | Nitrogen | nitrogen regulation and transport | cynS;cyanatelyase | EC:4.2.1.104 |
| K02006 | Metals | cobalt/nickel | cbiO;cobalt/nickeltransportsystemATP-bindingprotein |  |
| K02007 | Metals | cobalt/nickel | cbiM;cobalt/nickeltransportsystempermeaseprotein |  |
| K02008 | Metals | cobalt/nickel | cbiQ;cobalt/nickeltransportsystempermeaseprotein |  |
| K02009 | Metals | cobalt/nickel | cbiN;cobalt/nickeltransportprotein |  |
| K02010 | Metals | iron | afuC,fbpC;iron(III)transportsystemATP-bindingprotein | EC:7.2.2.7 |
| K02011 | Metals | iron | afuB,fbpB;iron(III)transportsystempermeaseprotein |  |
| K02012 | Metals | iron | afuA,fbpA;iron(III)transportsystemsubstrate-bindingprotein |  |
| K02017 | Metals | molybdenum | modC;molybdatetransportsystemATP-bindingprotein | EC:7.3.2.5 |
| K02018 | Metals | molybdenum | modB;molybdatetransportsystempermeaseprotein |  |
| K02019 | Metals | molybdenum | modE;molybdatetransportsystemregulatoryprotein |  |
| K02020 | Metals | molybdenum | modA;molybdatetransportsystemsubstrate-bindingprotein |  |
| K02045 | Sulfur | sulfur transport | cysA;sulfate/thiosulfatetransportsystemATP-bindingprotein | EC:7.3.2.3 |
| K02046 | Sulfur | sulfur transport | cysU;sulfate/thiosulfatetransportsystempermeaseprotein |  |
| K02047 | Sulfur | sulfur transport | cysW;sulfate/thiosulfatetransportsystempermeaseprotein |  |
| K02048 | Sulfur | sulfur transport | cysP;sulfate/thiosulfatetransportsystemsubstrate-bindingprotein |  |
| K02188 | Metals | cobalt | cbiD;cobalt-precorrin-5B(C1)-methyltransferase | EC:2.1.1.195 |
| K02189 | Metals | cobalt | cbiG;cobalt-precorrin5Ahydrolase | EC:3.7.1.12 |
| K02190 | Metals | nickel | cbiK;sirohydrochlorincobaltochelatase | EC:4.99.1.3 |
| K02191 | Metals | cobalt | cbiT;cobalt-precorrin-6B(C15)-methyltransferase | EC:2.1.1.196 |
| K02227 | Metals | cobalt | cbiB,cobD;adenosylcobinamide-phosphatesynthase | EC:6.3.1.10 |
| K02305 | Nitrogen | denitrification | norC;nitricoxidereductasesubunitC |  |
| K02362 | Metals | siderophore | entD;enterobactinsynthetasecomponentD | EC:6.3.2.142.7.8.- |
| K02363 | Metals | siderophore | entE,dhbE,vibE,mxcE;2,3-dihydroxybenzoate-AMPligase | EC:6.3.2.142.7.7.58 |
| K02364 | Metals | siderophore | entF;enterobactinsynthetasecomponentF | EC:6.3.2.14 |
| K02439 | Sulfur | sulfur transport | glpE;thiosulfatesulfurtransferase | EC:2.8.1.1 |
| K02567 | Nitrogen | nitrate reduction | napA;periplasmicnitratereductaseNapA | EC:1.7.99.- |
| K02568 | Nitrogen | nitrate reduction | napB;cytochromec-typeproteinNapB |  |
| K02575 | Nitrogen | nitrogen regulation and transport | NRT,narK,nrtP,nasA;MFStransporter,NNPfamily,nitrate/nitrite |  |
| K02586 | Nitrogen | nitrogen fixation | nifD;nitrogenasemolybdenum-ironproteinalphachain | EC:1.18.6.1 |
| K02588 | Nitrogen | nitrogen fixation | nifH;nitrogenaseironproteinNifH |  |
| K02591 | Nitrogen | nitrogen fixation | nifK;nitrogenasemolybdenum-ironproteinbetachain | EC:1.18.6.1 |
| K03322 | Metals | manganese | mntH;manganesetransportprotein |  |
| K03385 | Nitrogen | dissimilatory nitrate reduction | nrfA;nitritereductase(cytochromec-552) | EC:1.7.2.2 |
| K03388 | Methane | methanogenesis | hdrA2;heterodisulfidereductasesubunitA2 | EC:1.8.7.31.8.98.41.8.98.51.8.98.6 |
| K03389 | Methane | methanogenesis | hdrB2;heterodisulfidereductasesubunitB2 | EC:1.8.7.31.8.98.41.8.98.51.8.98.6 |
| K03390 | Methane | methanogenesis | hdrC2;heterodisulfidereductasesubunitC2 | EC:1.8.7.31.8.98.41.8.98.51.8.98.6 |
| K03399 | Metals | cobalt | cbiE;cobalt-precorrin-7(C5)-methyltransferase | EC:2.1.1.289 |
| K03421 | Methane | methanotrophy | mcrC;methyl-coenzymeMreductasesubunitC |  |
| K03422 | Methane | methanotrophy | mcrD;methyl-coenzymeMreductasesubunitD |  |
| K03795 | Metals | cobalt | cbiX;sirohydrochlorincobaltochelatase | EC:4.99.1.3 |
| K03813 | Metals | molybdenum | modD;molybdenumtransportprotein | EC:2.4.2.- |
| K04561 | Nitrogen | dissimilatory nitrate reduction | norB;nitricoxidereductasesubunitB | EC:1.7.2.5 |
| K04778 | Metals | siderophore | vibH;vibriobactinsynthetase |  |
| K04780 | Metals | siderophore | dhbF;nonribosomalpeptidesynthetaseDhbF |  |
| K04781 | Metals | siderophore | mbtI,irp9,ybtS;salicylatesynthetase | EC:5.4.4.24.2.99.21 |
| K04782 | Metals | siderophore | pchB;isochorismatepyruvatelyase | EC:4.2.99.21 |
| K04783 | Metals | siderophore | irp5,ybtE;yersiniabactinsalicyl-AMPligase | EC:6.3.2.- |
| K04784 | Metals | siderophore | irp2,HMWP2;yersiniabactinnonribosomalpeptidesynthetase |  |
| K04785 | Metals | siderophore | irp3,ybtU;yersiniabactinsynthetase,thiazolinylreductasecomponent |  |
| K04786 | Metals | siderophore | irp1,HMWP1;yersiniabactinnonribosomalpeptide/polyketidesynthase |  |
| K04787 | Metals | siderophore | mbtA;mycobactinsalicyl-AMPligase | EC:6.3.2.- |
| K04788 | Metals | siderophore | mbtB;mycobactinphenyloxazolinesynthetase |  |
| K04789 | Metals | siderophore | mbtE;mycobactinpeptidesynthetaseMbtE |  |
| K04790 | Metals | siderophore | mbtC;mycobactinpolyketidesynthetaseMbtC |  |
| K04791 | Metals | siderophore | mbtD;mycobactinpolyketidesynthetaseMbtD |  |
| K04792 | Metals | siderophore | mbtF;mycobactinpeptidesynthetaseMbtF |  |
| K04793 | Metals | siderophore | mbtG;mycobactinlysine-N-oxygenase |  |
| K05601 | Nitrogen | nitrification | hcp;hydroxylaminereductase | EC:1.7.99.1 |
| K05772 | Metals | tungsten | tupA,vupA;tungstatetransportsystemsubstrate-bindingprotein |  |
| K05773 | Metals | tungsten | tupB,vupB;tungstatetransportsystempermeaseprotein |  |
| K05776 | Metals | molybdenum | modF;molybdatetransportsystemATP-bindingprotein |  |
| K06857 | Metals | tungsten | tupC,vupC;tungstatetransportsystemATP-bindingprotein | EC:7.3.2.6 |
| K07306 | Sulfur | DMSO reduction | dmsA;anaerobicdimethylsulfoxidereductasesubunitA | EC:1.8.5.3 |
| K07307 | Sulfur | DMSO reduction | dmsB;anaerobicdimethylsulfoxidereductasesubunitB |  |
| K07308 | Sulfur | DMSO reduction | dmsC;anaerobicdimethylsulfoxidereductasesubunitC |  |
| K08260 | Metals | cobalt | cbiZ;adenosylcobinamidehydrolase | EC:3.5.1.90 |
| K08264 | Methane | methanogenesis | hdrD;heterodisulfidereductasesubunitD | EC:1.8.98.1 |
| K08265 | Methane | methanogenesis | hdrE;heterodisulfidereductasesubunitE | EC:1.8.98.1 |
| K09815 | Metals | zinc | znuA;zinctransportsystemsubstrate-bindingprotein |  |
| K09816 | Metals | zinc | znuB;zinctransportsystempermeaseprotein |  |
| K09817 | Metals | zinc | znuC;zinctransportsystemATP-bindingprotein | EC:7.2.2.- |
| K10094 | Metals | nickel | cbiK;nickeltransportprotein |  |
| K10535 | Nitrogen | nitrification | hao;hydroxylaminedehydrogenase | EC:1.7.2.6 |
| K10944 | Methane | methanotrophy | pmoA-amoA;methane/ammoniamonooxygenasesubunitA | EC:1.14.18.31.14.99.39 |
| K10945 | Methane | methanotrophy | pmoB-amoB;methane/ammoniamonooxygenasesubunitB |  |
| K10946 | Methane | methanotrophy | pmoC-amoC;methane/ammoniamonooxygenasesubunitC |  |
| K11180 | Sulfur | dissimilatory sulfate reduction | dsrA;dissimilatorysulfitereductasealphasubunit | EC:1.8.99.5 |
| K11181 | Sulfur | dissimilatory sulfate reduction | dsrB;dissimilatorysulfitereductasebetasubunit | EC:1.8.99.5 |
| K11260 | Methane | methanogenesis | fwdG;4Fe-4Sferredoxin |  |
| K11261 | Methane | methanogenesis | fwdE,fmdE;formylmethanofurandehydrogenasesubunitE | EC:1.2.7.12 |
| K11601 | Metals | manganese | mntC;manganesetransportsystemsubstrate-bindingprotein |  |
| K11602 | Metals | manganese | mntB;manganesetransportsystempermeaseprotein |  |
| K11603 | Metals | manganese | mntA;manganesetransportsystemATP-bindingprotein | EC:7.2.2.5 |
| K11604 | Metals | manganese/iron | sitA;manganese/irontransportsystemsubstrate-bindingprotein |  |
| K11605 | Metals | manganese/iron | sitC;manganese/irontransportsystempermeaseprotein |  |
| K11606 | Metals | manganese/iron | sitD;manganese/irontransportsystempermeaseprotein |  |
| K11607 | Metals | manganese/iron | sitB;manganese/irontransportsystemATP-bindingprotein |  |
| K11704 | Metals | iron/zinc/manganese/copper | mtsA;iron/zinc/manganese/coppertransportsystemsubstrate-bindingprotein |  |
| K11705 | Metals | iron/zinc/manganese/copper | mtsC;iron/zinc/manganese/coppertransportsystempermeaseprotein |  |
| K11706 | Metals | iron/zinc/manganese/copper | mtsB;iron/zinc/manganese/coppertransportsystemATP-bindingprotein |  |
| K11707 | Metals | manganese/zinc/iron | troA,mntA,znuA;manganese/zinc/irontransportsystemsubstrate-bindingprotein |  |
| K11708 | Metals | manganese/zinc/iron | troC,mntC,znuB;manganese/zinc/irontransportsystempermeaseprotein |  |
| K11709 | Metals | manganese/zinc/iron | troD,mntD,znuB;manganese/zinc/irontransportsystempermeaseprotein |  |
| K11710 | Metals | manganese/zinc/iron | troB,mntB,znuC;manganese/zinc/irontransportsystemATP-bindingprotein |  |
| K11924 | Metals | manganese | mntR;DtxRfamilytranscriptionalregulator,manganesetransportregulator |  |
| K12237 | Metals | siderophore | vibF;nonribosomalpeptidesynthetaseVibF |  |
| K12239 | Metals | siderophore | pchE;dihydroaeruginoicacidsynthetase |  |
| K12240 | Metals | siderophore | pchF;pyochelinsynthetase |  |
| K13255 | Metals | iron | fhuF;ferricironreductaseproteinFhuF |  |
| K13541 | Metals | cobalt | cbiGH-cobJ;cobalt-precorrin5Ahydrolase/precorrin-3BC17-methyltransferase | EC:3.7.1.122.1.1.131 |
| K13811 | Sulfur | assimilatory sulfate reduction | PAPSS;3'-phosphoadenosine5'-phosphosulfatesynthase | EC:2.7.7.42.7.1.25 |
| K13942 | Methane | methanogenesis | hmd;5,10-methenyltetrahydromethanopterinhydrogenase | EC:1.12.98.2 |
| K14080 | Methane | methanogenesis | mtaA; methyl-Co(III)methanol-specificcorrinoidprotein:coenzymeMmethyltransferase | EC:2.1.1.246 |
| K14081 | Methane | methanogenesis | mtaC;methanolcorrinoidprotein |  |
| K14126 | Methane | methanogenesis | mvhA,vhuA,vhcA;F420-non-reducinghydrogenaselargesubunit | EC:1.12.99.-1.8.98.5 |
| K14127 | Methane | methanogenesis | mvhD,vhuD,vhcD;F420-non-reducinghydrogenaseiron-sulfursubunit | EC:1.12.99.-1.8.98.5 |
| K14128 | Methane | methanogenesis | mvhG,vhuG,vhcG;F420-non-reducinghydrogenasesmallsubunit | EC:1.12.99.-1.8.98.5 |
| K15495 | Metals | molybdenum/tungsten | wtpA;molybdate/tungstatetransportsystemsubstrate-bindingprotein |  |
| K15496 | Metals | molybdenum/tungsten | wtpB;molybdate/tungstatetransportsystempermeaseprotein |  |
| K15497 | Metals | molybdenum/tungsten | wtpC;molybdate/tungstatetransportsystemATP-bindingprotein | EC:7.3.2.57.3.2.6 |
| K15576 | Nitrogen | nitrogen regulation and transport | nrtA,nasF,cynA;nitrate/nitritetransportsystemsubstrate-bindingprotein |  |
| K15577 | Nitrogen | nitrogen regulation and transport | nrtB,nasE,cynB;nitrate/nitritetransportsystempermeaseprotein |  |
| K15578 | Nitrogen | nitrogen regulation and transport | nrtC,nasD;nitrate/nitritetransportsystemATP-bindingprotein | EC:3.6.3.- |
| K15579 | Nitrogen | nitrogen regulation and transport | nrtD,cynD;nitrate/nitritetransportsystemATP-bindingprotein |  |
| K15653 | Metals | siderophore | mxcG;nonribosomalpeptidesynthetaseMxcG |  |
| K15864 | Nitrogen | denitrification | nirS;nitritereductase(NO-forming)/hydroxylaminereductase | EC:1.7.2.11.7.99.1 |
| K15876 | Nitrogen | dissimilatory nitrate reduction | nrfH;cytochromecnitritereductasesmallsubunit |  |
| K16091 | Metals | iron | fecA;Fe(3+)dicitratetransportprotein |  |
| K16157 | Methane | methanotrophy | mmoX;methanemonooxygenasecomponentAalphachain | EC:1.14.13.25 |
| K16158 | Methane | methanotrophy | mmoY;methanemonooxygenasecomponentAbetachain | EC:1.14.13.25 |
| K16159 | Methane | methanotrophy | mmoZ;methanemonooxygenasecomponentAgammachain | EC:1.14.13.25 |
| K16160 | Methane | methanotrophy | mmoB;methanemonooxygenaseregulatoryproteinB |  |
| K16161 | Methane | methanotrophy | mmoC;methanemonooxygenasecomponentC | EC:1.14.13.25 |
| K16162 | Methane | methanotrophy | mmoD;methanemonooxygenasecomponentD |  |
| K16915 | Metals | nickel | cbiL;nickeltransportprotein |  |
| K16950 | Sulfur | sulfite reduction | asrA;anaerobicsulfitereductasesubunitA |  |
| K16951 | Sulfur | sulfite reduction | asrB;anaerobicsulfitereductasesubunitB |  |
| K16952 | Sulfur | sulfur oxidation | sor;sulfuroxygenase/reductase | EC:1.13.11.55 |
| K17222 | Sulfur | thiosulfate oxidation | soxA;L-cysteineS-thiosulfotransferase | EC:2.8.5.2 |
| K17223 | Sulfur | thiosulfate oxidation | soxX;L-cysteineS-thiosulfotransferase | EC:2.8.5.2 |
| K17224 | Sulfur | thiosulfate oxidation | soxB;S-sulfosulfanyl-L-cysteinesulfohydrolase | EC:3.1.6.20 |
| K17225 | Sulfur | thiosulfate oxidation | soxC;sulfanedehydrogenasesubunitSoxC |  |
| K17226 | Sulfur | thiosulfate oxidation | soxY;sulfur-oxidizingproteinSoxY |  |
| K17227 | Sulfur | thiosulfate oxidation | soxZ;sulfur-oxidizingproteinSoxZ |  |
| K17229 | Sulfur | sulfide oxidation | fccB;sulfidedehydrogenase flavocytochromecflavoproteinchain | EC:1.8.2.3 |
| K17230 | Sulfur | sulfide oxidation | fccA;cytochromesubunitofsulfidedehydrogenase |  |
| K17725 | Sulfur | thiosulfate oxidation | ETHE1;sulfurdioxygenase | EC:1.13.11.18 |
| K17993 | Sulfur | sulfur reduction | hydA;sulfhydrogenasesubunitalpha | EC:1.12.1.31.12.1.5 |
| K17994 | Sulfur | sulfur reduction | hydD;sulfhydrogenasesubunitdelta | EC:1.12.1.31.12.1.5 |
| K17995 | Sulfur | sulfur reduction | hydG;sulfhydrogenasesubunitgamma(sulfurreductase) | EC:1.12.98.4 |
| K17996 | Sulfur | sulfur reduction | hydB;sulfhydrogenasesubunitbeta(sulfurreductase) | EC:1.12.98.4 |
| K19611 | Metals | iron | fepA,pfeA,iroN,pirA;ferricenterobactinreceptor |  |
