# Supplementary material for: Variations and gradients between methane seep and off-seep microbial communities in a submarine canyon system in the Northeast Pacific
Source: PeerJ. 2023 Mar 28;11:e15119. doi: 10.7717/peerj.15119 (PMC10064993; doi:10.7717/peerj.15119)
Supplement: Supplemental Information 4 [file peerj-11-15119-s004.docx]

***Supplemental Table 3 – ANCOM analysis statistical values, including log-fold changes, p-values, and W statistics.***

| ***Non-seep vs. Dagorlad*** | | | |
| --- | --- | --- | --- |
| **Family** | **Log-fold change** | **W** | **p-value** |
| Sulfurovaceae | 6.07370 | 22.8514 | 1.42E-115 |
| Sulfurimonadaceae | 5.46631 | 22.2625 | 8.54E-110 |
| Desulfobulbaceae | -0.73517 | -1.7605 | 7.83E-02 |
| Desulfosarcinaceae | -0.01054 | -0.0676 | 9.46E-01 |
| Desulfocapsaceae | 2.59805 | 13.0391 | 7.33E-39 |
| ANME-1a | 0.09815 | 0.3836 | 7.01E-01 |
| ANME-1b | 0.19182 | 1.3370 | 1.81E-01 |
| ANME-2a-2b | 3.87506 | 9.7008 | 2.99E-22 |
| ANME-2c | 2.74145 | 4.8240 | 1.41E-06 |
| Scalinduaceae | -2.88668 | -7.6522 | 1.98E-14 |
| Woeseiaceae | -2.19372 | -4.9160 | 8.83E-07 |
| NB1-j | -2.65411 | -9.1752 | 4.51E-20 |
| Nitrosococcaceae | -1.89485 | -4.6625 | 3.12E-06 |
| ***Non-seep vs. Emyn Muil*** | | | |
| **Family** | **Log-fold change** | **W** | **p-value** |
| Sulfurovaceae | 4.58917 | 10.6741 | 1.35E-26 |
| Sulfurimonadaceae | 3.47081 | 7.0005 | 2.55E-12 |
| Desulfobulbaceae | -2.09868 | -4.6474 | 3.36E-06 |
| Desulfosarcinaceae | 0.44804 | 2.8179 | 4.83E-03 |
| Desulfocapsaceae | 1.54767 | 3.2459 | 1.17E-03 |
| ANME-1a | 4.28288 | 8.9171 | 4.79E-19 |
| ANME-1b | 3.89465 | 6.8308 | 8.45E-12 |
| ANME-2a-2b | 4.01878 | 10.7576 | 5.46E-27 |
| ANME-2c | 2.35716 | 7.2589 | 3.90E-13 |
| Scalinduaceae | -4.52319 | -16.7223 | 9.02E-63 |
| Woeseiaceae | -3.41942 | -8.0148 | 1.10E-15 |
| NB1-j | -4.48378 | -16.7577 | 4.97E-63 |
| Nitrosococcaceae | -3.41162 | -10.6295 | 2.17E-26 |
| ***Non-seep vs. Westmarch*** | | | |
| **Family** | **Log-fold change** | **W** | **p-value** |
| Sulfurovaceae | 7.85031 | 32.1262 | 1.90E-226 |
| Sulfurimonadaceae | 5.59316 | 27.0897 | 1.30E-161 |
| Desulfobulbaceae | 1.51889 | 6.5873 | 4.48E-11 |
| Desulfosarcinaceae | 0.35635 | 1.8489 | 6.45E-02 |
| Desulfocapsaceae | 4.47010 | 20.0012 | 5.38E-89 |
| ANME-1a | -0.40227 | -1.2264 | 2.20E-01 |
| ANME-1b | 0.94570 | 2.5289 | 1.14E-02 |
| ANME-2a-2b | 4.71027 | 10.8069 | 3.19E-27 |
| ANME-2c | 0.35293 | 1.0281 | 3.04E-01 |
| Scalinduaceae | -1.74912 | -7.0711 | 1.54E-12 |
| Woeseiaceae | -0.75751 | -3.4413 | 5.79E-04 |
| NB1-j | -1.20578 | -4.4468 | 8.72E-06 |
| Nitrosococcaceae | -0.70481 | -2.3503 | 1.88E-02 |
