# Supplementary material for: Variations and gradients between methane seep and off-seep microbial communities in a submarine canyon system in the Northeast Pacific
Source: PeerJ. 2023 Mar 28;11:e15119. doi: 10.7717/peerj.15119 (PMC10064993; doi:10.7717/peerj.15119)
Supplement: Supplemental Information 5 [file peerj-11-15119-s005.docx]

***Supplemental Table 4 – Continued SIMPER analysis of genes contributing to up to 40% of variance between seep and non-seep sites.***

| **KO** | **Cumulative % Variance** | **Process** | **More abundant in…** | **Gene** |
| --- | --- | --- | --- | --- |

| *accounting for up to 40% of variance:* | | | | |
| --- | --- | --- | --- | --- |
| K02006 | 21.61% | cobalt/nickel transport | Seep | cbiO; cobalt/nickel transport system ATP-binding protein |
| K02012 | 23.11% | iron transport | Non-seep | afuA,fbpA; iron(III) transport system substrate-binding protein |
| K14127 | 24.60% | methanotrophy | Seep | mvhD,vhuD,vhcD; F420-non-reducing hydrogenase iron-sulfur subunit |
| K02011 | 26.08% | iron transport | Non-seep | afuB,fbpB; iron(III) transport system permease protein |
| K09816 | 27.54% | zinc transport | Seep | znuB; zinc transport system permease protein |
| K02010 | 29.00% | iron transport | Non-seep | afuC,fbpC; iron(III) transport system ATP-binding protein |
| K02575 | 30.43% | nitrogen regulation/ transport | Non-seep | NRT,narK,nrtP,nasA; MFS transporter, NNP family, nitrate/nitrite |
| K00958 | 31.85% | sulfate reduction | Seep | sat,met3; sulfateadenylyltransferase |
| K02009 | 33.26% | cobalt/nickel transport | Seep | cbiN; cobalt/nickel transport protein |
| K00381 | 34.60% | assimilatory sulfate reduction | Non-seep | cysI; sulfite reductase(NADPH) hemoprotein beta-component |
| K15497 | 35.94% | molybdenum/ tungsten transport | Seep | wtpC; molybdate/tungstate transport system ATP-binding protein |
| K02008 | 37.24% | cobalt/nickel transport | Seep | cbiQ; cobalt/nickel transport system permease protein |
| K02007 | 38.54% | cobalt/nickel transport | Seep | cbiM; cobalt/nickel transport system permease protein |
| K02189 | 39.79% | cobalt transport | Seep | cbiG; cobalt-precorrin 5A hydrolase |
| K02227 | 41.01% | cobalt transport | Seep | cbiB,cobD; adenosyl cobinamide-phosphate synthase |
